# Transcriptomic Profiling of Spleen in Grass-Fed and Grain-Fed Angus Cattle

**DOI:** 10.1371/journal.pone.0135670

**Published:** 2015-09-14

**Authors:** Yaokun Li, José A. Carrillo, Yi Ding, Yanghua He, Chunping Zhao, Jianan Liu, George E. Liu, Linsen Zan, Jiuzhou Song

**Affiliations:** 1 College of Animal Science and Technology, Northwest A&F University, Yangling, Shaanxi, 712100, P. R. China; 2 Department of Animal & Avian Sciences, University of Maryland, College Park, Maryland, 20742, United States of America; 3 Animal Genomics and Improvement Laboratory, USDA-ARS, Building 306, Room 111, BARC-East, Beltsville, Maryland, 20705, United States of America; Chang Gung University, TAIWAN

## Abstract

The grass-fed cattle obtain nutrients directly from pastures containing limited assimilable energy but abundant amount of fiber; by contrast, grain-fed steers receive a diet that is comprised mainly of grains and serves as an efficient source of high-digestible energy. Besides energy, these two types of diet differ in a large number of nutritional components. Additionally, animals maintained on rich-energy regimen are more likely to develop metabolic disorders and infectious diseases than pasture raised individuals. Thus, we hypothesize that spleen–a relevant immune organ–may function differently under disparate regimes. The objective of this study was to find the differentially expressed genes in the spleen of grass-fed and grain-fed steers, and furtherly explore the potential involved biopathways. Through RNA sequencing (RNA-Seq), we detected 123 differentially expressed genes. Based on these genes, we performed an Ingenuity Pathway Analysis (IPA) and identified 9 significant molecular networks and 13 enriched biological pathways. Two of the pathways, Nur77 signaling in T lymphocytes and calcium-induced T lymphocyte apoptosis which are immune related, contain a pair of genes HLA-DRA and NR4A1 with dramatically altered expression level. Collectively, our results provided valuable insights into understanding the molecular mechanism of spleen under varied feeding regimens.

## Background

In many countries, cattle constitute a main source of protein and other nutrients, such as lipids and minerals. Among several breeds specialized for meat production, Aberdeen Angus stands out for its extensive contribution to the beef industry, especially in the United States. Using this breed, numerous and valuable research has been performed to improve growth and meat quality traits. Interestingly, many studies demonstrated that different feeding regimens could alter the nutritional composition of beef. Stearic acid, the only saturated fatty acid exerting impact on serum cholesterol, displays higher level in grass finished beef compared with grain-fed beef [1]. Several investigators also reported significant differences in omega-3: omega-6 ratio between grass-fed and grain-fed cattle [2,3]. Beta-carotene, the precursor of vitamin A, is significantly higher in muscle of pasture-fed steers compared to grain-fed animals [4]. Additionally, vitamin A could contribute for the integrity of mucous membrane and skin, protecting individuals against bacterial and viral infection [5,6]. Meanwhile, vitamin A could also modulate immune function through influencing white blood cells [7,8]. Several studies suggested that the amount of α-tocopherol (the most biologically active form of vitamin E) is higher in the meat of cattle finished on pasture than the grain-fed cattle [9–11]. Moreover, it was demonstrated that the variation of the dietary protein concentration could influence the expression of the immune genes, altering the immunological system [12,13]. It also has been reported that high-energy diets fed to maximize individuals’ performance could lead to higher incidence of metabolic disorders [14]. Therefore, grain-fed cattle suffer stronger metabolic stress than pasture-fed steers; and they tend to easily have respiratory and infectious disease.

Spleen–the largest lymphatic organ in the body–plays a critical role in the immune system. It constitutes the main filter of the body for blood-borne antigens and pathogens, as well as an important organ for erythrocyte homeostasis and iron metabolism [15]. The spleen is composed of red and white pulp. The white pulp contains B cell and T cell zones, and generates antigen-specific immune response protecting individuals against viral, bacterial and fungal infection. The red pulp mainly filters blood and regulates iron recycling from aged red blood cells. However, limited information about spleen transcriptome has been reported and the molecular mechanism of bovine spleen remains largely unknown.

In this project, we hypothesized that the spleen transcriptome might exhibit distinct characteristics under grass-fed and grain-fed regimes, which could result in different spleen function, especially affecting the immune response. To test it, we sampled spleen tissues from grass-fed and grain-fed Angus steers and performed a comparative study of gene expression using RNASeq method. Then, based on the differentially expressed genes (DEGs), we implemented a functional analysis and identified potential mechanisms that could contribute to the difference observed between both groups.

## Materials and Methods

### Sample collection

We collected spleen samples from two randomly chosen animals per group, totaling four samples. The animals were born and raised at the Wye Angus farm, which has produced genetically similar progenies. The genetic resemblance among individuals permitted us to better control the variation between experimental individuals, constituting an excellent resource to perform scientific research. All animals included in this study received the same diet until weaning. Next, we assigned the animals to one certain diet at random, and exclusively raised them under that regimen until termination. The diet of grain-fed group consisted of soybean, shelled corn, corn silage and trace minerals. The grass-fed steers normally received alfalfa harvested from land without any fertilizers, pesticides or other chemicals; during wintertime, bailage was supplied. Grass-fed individuals ate no animal, agricultural or industrial byproducts and never consumed any type of grain. Grain–fed animals reached the market weight around 14 month-old; however, grass-fed steers needed approximately 200 additional days to achieve the same weight. Immediately after termination at the Old Line Custom Meat Company (Baltimore, MD), a small piece of spleen was incised, washed and frozen at -80°C for posterior processing.

### RNA extraction and sequencing

We extracted total RNA individually (two animals per group) by using Trizol (Invitrogen, Carlsbad, CA, USA) followed by DNase digestion; Qiagen RNeasy column was utilized for purification (Invitrogen, Carlsbad, CA, USA). RNA samples were dissolved in RNAse-free H_2_O, and checked for integrity and quality with a NanoDrop 1000 spectrophotometer and a 1.5% agarose gel. The samples were labeled through adding 6-bp adaptors and then pooled for sequencing. Finally, the RNA-seq library was sequenced using an Illumina HiSeq 2000 device, as described previously [16].

### Data analysis and bioinformatics

First, we checked the quality of the raw reads through FastQC, which is an online tool that thoroughly examines the reads and generates a detailed and extensive quality assurance report [17]. Then, employing Bowtie (Ultrafast, memory-efficient short read aligner), we aligned the reads to the reference genome (Bos_taurus_UMD3.1/bosTau6) downloaded from the UCSC (http://genome.ucsc.edu/). During this step, we trimmed the first 15 bases of each read (50 bp) to avoid low Phred quality scores, which resulted in 35 bp tags. Via the summarizeOverlaps function implemented in R, we executed the reads counting for each gene. Subsequently, we identified the differentially expressed genes using the generalized linear model (GLM) approach included in the edgeR software package, which required a designed matrix to describe the two treatment conditions grass-fed and grain-fed. The edgeR package estimated an effective library size applying a scaling factor based on the library size. The normalization in this approach is model-based and the original counts remain the same without any type of transformation. For variance calculation, we first estimated a common dispersion for all reads and then forced the tagwise variation towards the common dispersion based on a Bayesian strategy, which resulted in higher sensitivity for detection. Finally, we applied the false discovery rate (FDR) of <0.1 as a threshold to call the genes with different expression levels.

After we obtained the DEGs list, we performed a GO enrichment analysis and examined the biological processes, cellular components and molecular functions associated with those DEGs through the online software DAVID Bioinformatics Resources 6.7 [18]. Fisher’s exact test was used to determine the enrichment of the GO terms. Additionally, we identified the enriched networks, molecular functions and pathways in the Ingenuity Pathways Analysis (IPA, Ingenuity Systems, and www.ingenuity.com) platform, which was a highly convenient application [19–21]. During the IPA analysis, the p-value obtained via Fisher’s exact test was used as a significance criterion.

### Quantitative real-time polymerase chain reaction (qRT-PCR) analysis

Through qRT-PCR on the iCycler iQ PCR system (Bio-Rad, Hercules, CA, USA), we validated and compared the expression of 9 randomly selected DEGs from the RNA-Seq analysis. We obtained the template cDNA by employing the iScript First Strand Synthesis System Kit (Bio-Rad) for reverse transcription PCR with 500 ng of total RNA. The qRT-PCR reactions were performed with a QuantiTect SYBR Green PCR Kit (Qiagen, Valencia, CA, USA) according to the manufacturer’s instructions. We designed the primers using an online tool (http://frodo.wi.mit.edu/primer3/); the primer sequences are provided in S1 Table. We chose GAPDH as the control gene [22]. For each sample, we performed three technical and two independent biological replicates.

## Results

### Alignment of Reads and Gene Expression Analysis

**Fig 1:**
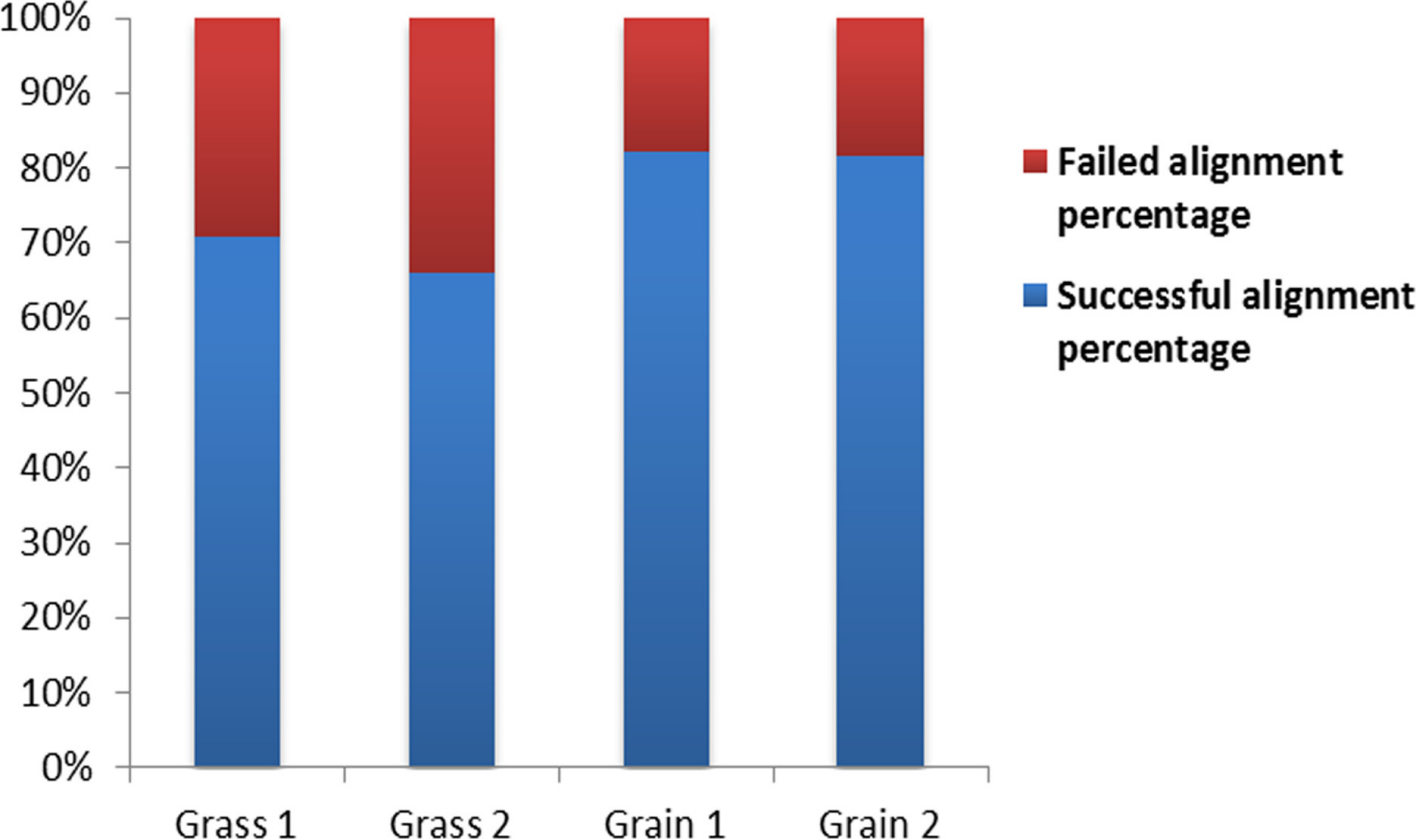
Alignment level of RNA-Seq reads to the Bovine Genome.

**Fig 2:**
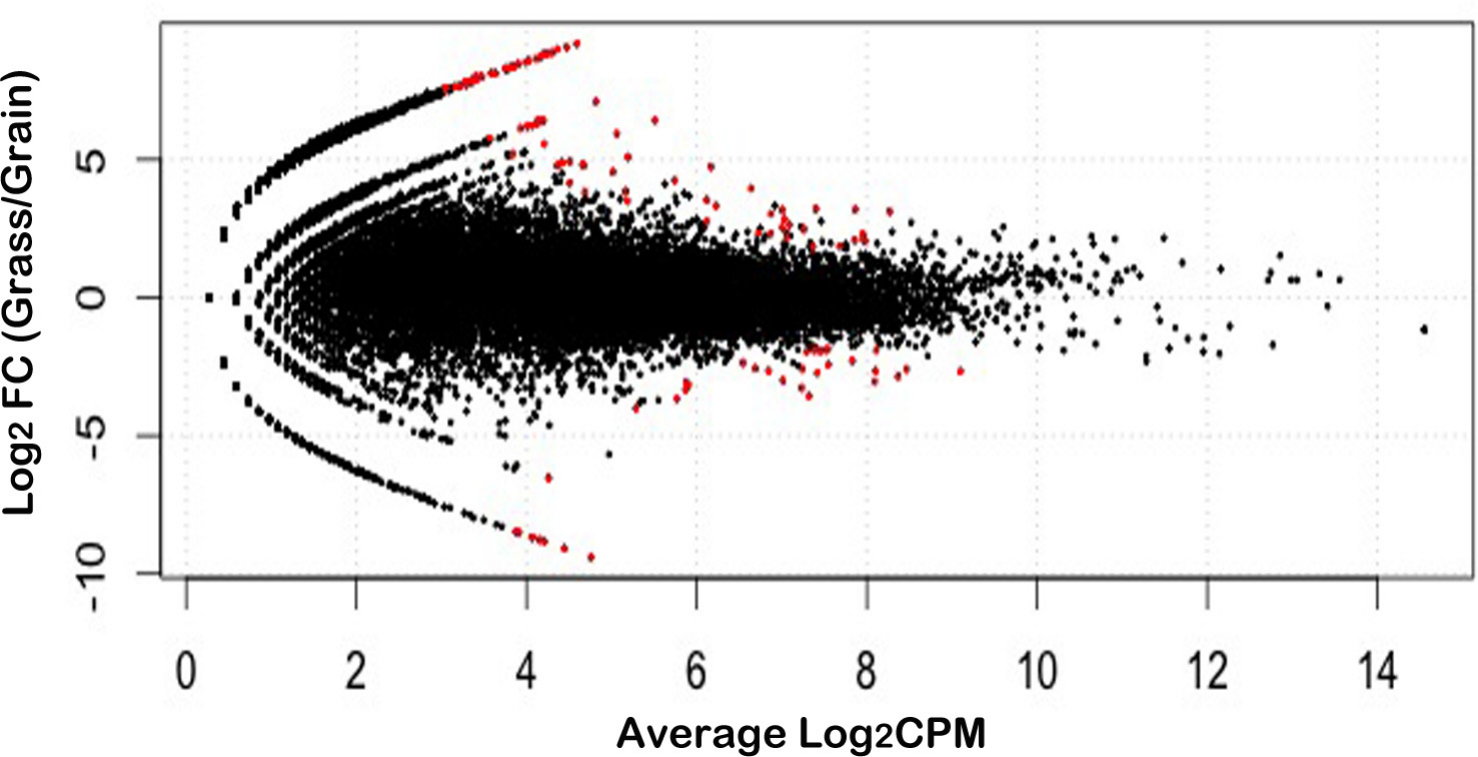
Differentially expressed spleen genes between grass-fed and grain-fed steers. MA-plot was obtained from two independent biological replicates at an FDR of 0.1. The black points represent the genes without expression difference. FC means fold-change. CPM means counts per million.

We totally had four experimental samples; the alignment levels were 70.73%, 65.92%, 82.10% and 81.54%, respectively (Fig 1). For the statistical analysis, we applied the edgeR package implemented in R to detect the genes with divergent expression profiles in the spleen of grass-fed and grain-fed steers. The threshold of FDR <0.1 was used to call the significant difference. Following this strategy, we finally found 123 genes with distinct expression levels in both groups (Fig 2). From those genes, we found that 87 were highly expressed and the other 36 genes were down regulated in the spleen of grass-fed bovines compared with the grain-fed group. According to the log_2_FC ≥5 criteria, 52 genes in grass-fed steers spleen expressed higher than in grain finished group and only 9 genes increased their activity in the grain-fed animals. The top 10 DEGs in the spleen of both groups can be seen in Table 1. Among these genes, the expression levels of ENDOU, CD9, TMEM45B, SLC4A9, HADH, OVOL1 and FGFBP1 in grass-fed animals exceeded the levels observed in the grain-fed group, whereas the expression abundance of the genes LOC534630, RBMX2 and DOCK6 diminished in grass-fed steers. The complete list of DEGs between these two groups can be found in S2 Table.

**Table 1:** Top 10 differentially expressed genes in the spleen of grass-fed and grain-fed Angus Cattle.

Ensemble Gene ID	Symbol	Log_2_ FC(grass/grain)	FDR
ENSBTAG00000006252	LOC534630	-3.56744	5.72×10^−5^
ENSBTAG00000014771	RBMX2	-3.03993	1.24×10^−4^
ENSBTAG00000012275	ENDOU	6.41496	1.98×10^−4^
ENSBTAG00000014764	CD9	3.95444	1.09×10^−3^
ENSBTAG00000017602	TMEM45B	9.20076	1.58×10^−3^
ENSBTAG00000021775	SLC4A9	8.99572	1.58×10^−3^
ENSBTAG00000002049	HADH	3.20592	1.67×10^−3^
ENSBTAG00000012656	OVOL1	9.07147	1.80×10^−3^
ENSBTAG00000009569	DOCK6	-2.65744	2.06×10^−3^
ENSBTAG00000031497	FGFBP1	7.08732	2.52×10^−3^

### Validation of DEGs

**Fig 3:**
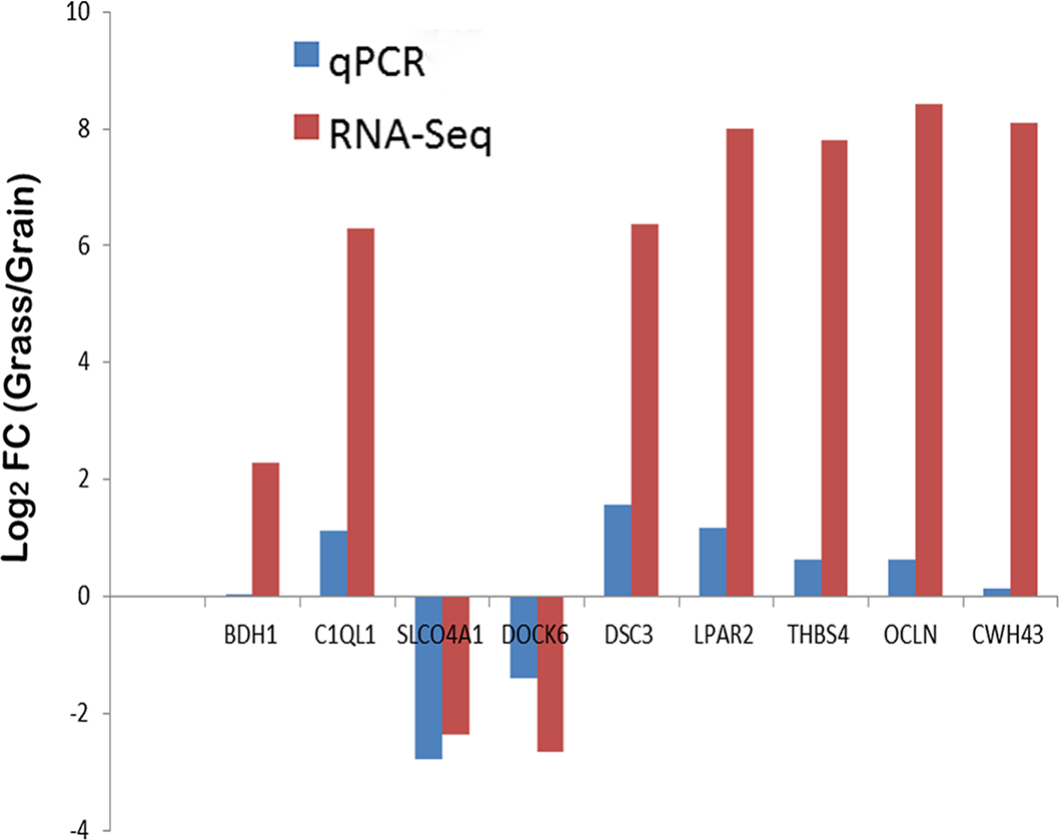
Validation of differentially expressed genes. The mean value of log2 (fold-change) for each group was compared in the bar chart for the 9 selected genes. qPCR data was normalized by GAPDH expression for each sample. Means of significant (FDR≤0.1) fold changes were computed for qPCR and DESeq using sample from the same 4 animals in each analysis. FC means fold-change.

We used qRT-PCR to confirm the expression level of 9 genes, which were randomly selected from the DEGs list. Through contrasting the qRT-PCR result with the RNA-Seq analysis, we found 100% consistency (Fig 3). Genes SLCO4A1 and DOCK6 were dramatically over expressed in grain-fed spleen. The other 7 genes showed less activity in grain finished steers compared with the grass-finished group. Collectively, the qRT-PCR validation of those 9 genes proved the accuracy of the RNA-Seq analysis.

### Gene Ontology Analysis

We used the online software DAVID (version 6.7) to perform the Gene Ontology (GO) enrichment analysis and identify functional characteristics of the DEGs discovered in our present study. We primarily focused on biological process, cellular component and molecular function remarkably enriched with the gene set. The result could be seen in Table 2. The most significant GO terms were peptide cross-linking in biological process, plasma membrane part for cellular component and growth factor binding in molecular function. Most of the biological processes are related to development, adhesion and membrane transport. For cellular component, the GO terms enriched with the DEGs have large influence on plasma membrane and cell junction. However, in terms of molecular function, we only found one significant GO term: growth factor binding.

**Table 2:** Gene Ontology (GO) terms enriched with differentially expressed genes (P<0.05).

GO terms	Observed*	P	FDR
***Biological process***			
GO:0018149~peptide cross-linking	3	0.00409	0.89321
GO:0022610~biological adhesion	7	0.00744	0.86978
GO:0007155~cell adhesion	7	0.00744	0.86978
GO:0006811~ion transport	8	0.02458	0.98922
GO:0060073~micturition	2	0.02814	0.97969
GO:0046903~secretion	4	0.02995	0.96387
GO:0015672~monovalent inorganic cation transport	5	0.03012	0.93815
GO:0008544~epidermis development	3	0.03150	0.91762
GO:0007398~ectoderm development	3	0.03514	0.91294
***Cellular component***			
GO:0044459~plasma membrane part	14	0.00100	0.08851
GO:0005886~plasma membrane	18	0.00208	0.09247
GO:0005911~cell-cell junction	5	0.00399	0.11648
GO:0030054~cell junction	6	0.01322	0.26613
GO:0034702~ion channel complex	4	0.03040	0.43681
***Molecular function***			
GO:0019838~growth factor binding	3	0.03435	0.99883

*Number of the differentially expressed genes in the category

### Ingenuity Pathway Analysis

We employed the Ingenuity Pathway Analysis (IPA) online software to detect the canonical pathways involving the DEGs. The thirteen most significant ingenuity canonical pathways can be seen in Table 3. A large proportion of the pathways acted on biosynthesis and the function of T lymphocyte, which would provide valuable insights into explaining the molecular mechanism of spleen function.

**Table 3:** Canonical pathways enriched with differentially expressed genes by Ingenuity Pathway Analysis (IPA) (P < 0.05)

Ingenuity Canonical Pathways	Observed*	P value	FDR
Ketogenesis	2	0.0011	0.1275
Ubiquinol-10 Biosynthesis (Eukaryotic)	2	0.0016	0.0930
Retinoate Biosynthesis I	2	0.0118	0.4602
Retinoate Biosynthesis II	1	0.0199	0.5821
Branched-chain α-keto acid Dehydrogenase Complex	1	0.0199	0.4657
Ceramide Biosynthesis	1	0.0248	0.4836
Pregnenolone Biosynthesis	1	0.0297	0.4964
Nur77 Signaling in T Lymphocytes	2	0.0332	0.4856
eNOS Signaling	3	0.0339	0.4407
Epithelial Adherens Junction Signaling	3	0.0370	0.4329
Calcium-induced T Lymphocyte Apoptosis	2	0.0409	0.4350
Ketolysis	1	0.0442	0.4310
Histidine Degradation VI	1	0.0442	0.3978

*Number of the differentially expressed genes in the category

### Molecular subnetwork

**Fig 4:**
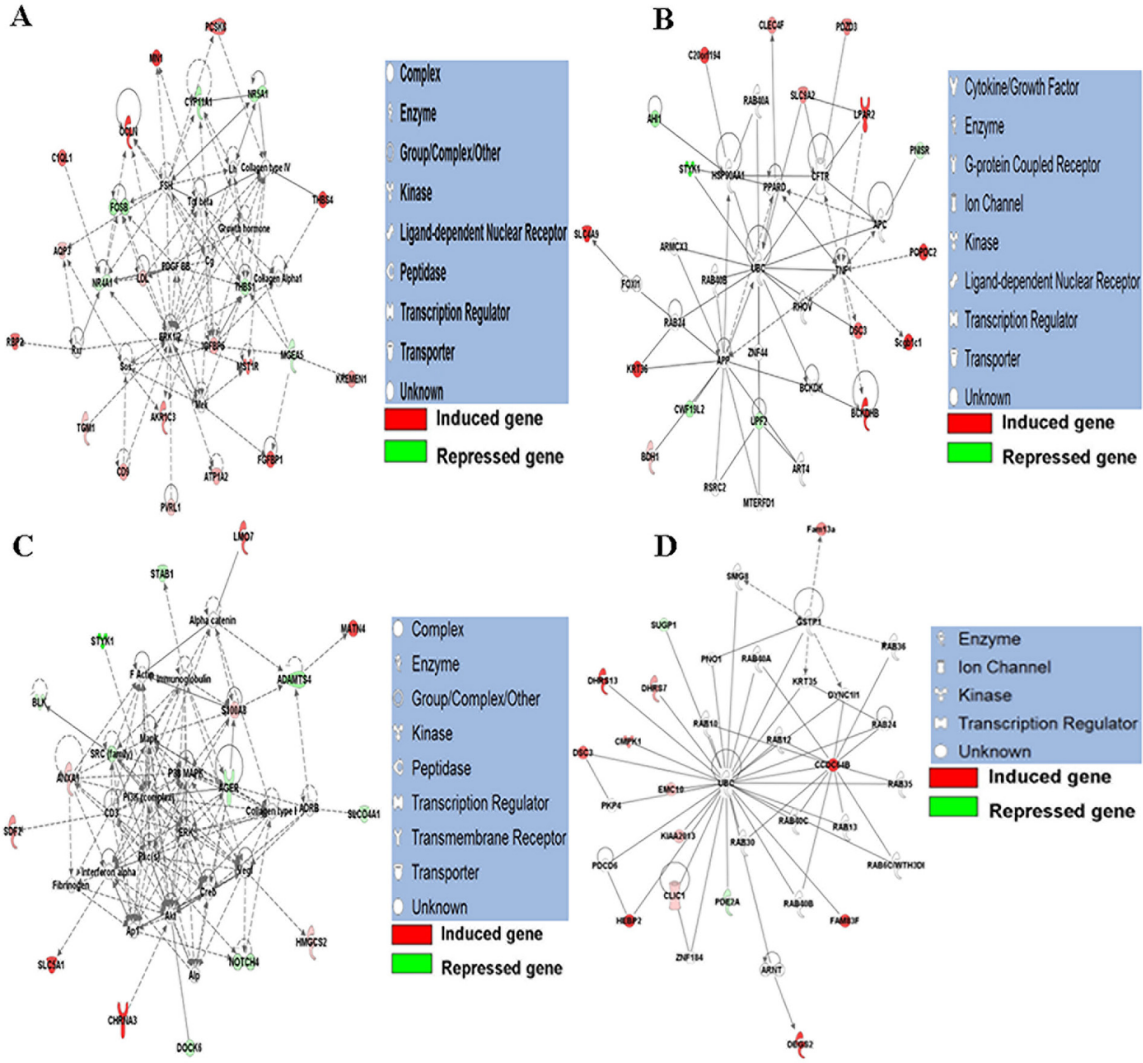
The top four molecule networks identified by Ingenuity Pathway Analysis (IPA). A: The most significant molecular network identified IPA. B: The second most significant molecular network identified by IPA. C: The third most significant molecular network identified by IPA. D: The fourth most significant molecular network identified by IPA.

We can examine the gene networks to predict the participation of other interacting molecules in the pathways. Those molecules might also play diverse key roles in spleen function. To assess this, we performed Fisher’s exact test based on the IPA molecular library, and found a total of 9 significant molecular networks (S3 Table). Fig 4 shows the 4 most remarkable networks. The first network (Fig 4A) consists of 22 DEGs and its most essential functions constitute cancer, cell morphology and cell death, and survival. The second network includes 17 DEGs and the functions of cancer, cell-to-cell signaling and interaction, respiratory system development and function are enriched in this structure (Fig 4B). The main functions of the third network involve cancer, inflammatory disease and connective tissue disorders; 16 genes from the DEGs list participate in this system (Fig 4C). The fourth network, which incorporates 14 DEGs, principally relates to cancer, nucleic acid metabolism, organismal injury and abnormalities (Fig 4D).

## Discussion

Since the appearance of organic products, the reality about their benefits and disadvantages has become controversial. Thus numerous studies have been performed to detect the true difference between organic and conventional food, and the possible consequence for the human and animal health [23–25]. Transcriptome sequencing constitutes an effective method to estimate gene expression through counting extensive number of sequenced reads [26]. Transcriptome analysis of different species including *Caenorhabditis elegans*, yeast, plants and mammals has been completed for diverse purposes [27–30]. However, transcriptomic data for spleen of bovines under different diets has not been disclosed. Considering that different feeding regimes would contribute to disparate probabilities in getting metabolic and infectious disease, and that spleen constitutes one of the most important immune organs; we expected to observe the DEGs associated with immune response as a result of distinct diets. The identification of those genes will lead to a better understanding of the functional mechanism of spleen in particular conditions. Several investigators have reported that the levels of the precursor of Vitamin A and E and cancer fighting antioxidants, including glutathione and superoxide dismutase activity, were different between pasture-fed and grain-fed beef [31,32]. Additionally, previous studies suggested that modification of the protein abundance in the diet could alter the expression of immune-related genes, further regulating and reshaping the immune response [12,13].

Studies on chicken showed that 177 genes exhibited different expression levels in the spleen after *Salmonella enteritidis* infection; among these genes, 99 were down-regulated and the other 78 were up-regulated [33]. In our present study, we detected 123 DEGs between grass-fed and grain-fed spleen of Angus cattle. In the grass-finished group, 87 were up-regulated while the other 36 decreased their gene activity. Regarding the top 10 most statistically significant genes, CD9 encodes a member of the transmembrane 4 superfamily and associates with lymphoid tissue structure and development, immune cell trafficking, hematopoiesis, lipid metabolism and inflammatory response; studies suggested that CD9 congregating at the T-cell side of the immunological synapse (IS) participates in the immune response and might support the integrin-mediated signaling at the IS [34,35]. SLC4A9, DOCK6 and FGFBP1 mainly interact with cancer. TMEM45B also showed altered expression level; however, limited information about TMEM45B existed; we hypothesized that this might be a potential gene to regulate the function of spleen. In our DEGs list, we also detected the same gene Notch4, which was discovered as the differentially expressed gene in the developmental process of spleen [36]. This gene is one of the members of Notch signaling pathway; it can regulate cell fate decisions and inhibit endothelium apoptosis [37].

Via GO enrichment analysis, we identified the genes CHRNA3, CLIC1, SLC4A9, SLC9A2, SLC5A1 and ATP1A2 which participate in the process of iron transport exerting effects on bacterial virulence and host immune response [38]. We also found a large number of genes functioning in the plasma membrane; four genes CHRNA3, CLIC1, KCTD1 and KCNMA1 were discovered in iron channel complex; except KCTD1, all others were associated with cancer. Meanwhile, the IPA analysis unveiled that HLA-DRA and NR4A1 were integrated into the Nur77 Signaling in T Lymphocytes pathway. These two genes were also associated with the calcium-induced T lymphocyte apoptosis pathway, which could exert effects on immunization. Additionally, several studies reported that HLA-DRA could affect tumors and NR4A1 has been correlated with various carcinomas [39,40]. Although considerable research has examined these two genes, no literature addressing their functions in the spleen has been published. Accordingly, it might be interesting to perform functional experiment of these genes on spleen to better understand their function in the immunological system.

Our results provide general information regarding the molecular mechanism and functional difference of spleen under variable feeding regimes. However, we recognized that our study suffered some limitations. Identification of the DEGs and the follow-up pathway/network analysis were conducted merely relying on the computational strategy; extensive experimental validation work is still needed. Therefore, overexpression and inhibition of relevant genes should be advisable for functional validation of our findings.

## Conclusions

In this study, we identified the genes and pathways that may influence the function of spleen in different diets. Totally, 123 DEGs were discovered between grass-fed and grain-fed cattle. According to those DEGs, 13 significant molecular networks involved in cancer, inflammatory and respiratory disease, were found in the IPA system. Most of the pathways enriched with the DEGs were associated with T lymphocyte and biosynthesis. In conclusion, our results contributed insights into understanding the mechanism of spleen to strengthen the disease resistance of animals.

## Supporting Information

S1 Table Primers used for quantitative real-time PCR validation. (XLS)

S2 Table Differentially expressed genes in the spleen of grass-fed and grain-fed cattle at a strict false discovery rate (FDR) <0.1. (XLS)

S3 Table The 8 most significant molecular networks found by Fisher’s exact test in the IPA system. (XLS)
